# Prevalence of Hepatitis ‘B’ and Hepatitis ‘C’ among preoperative cataract patients in Karachi

**DOI:** 10.1186/1756-0500-5-492

**Published:** 2012-09-06

**Authors:** Syed Saad Naeem, Efaza Umar Siddiqui, Abdul Nafey Kazi, SumaiyaTauseeq Khan, Farhan E Abdullah, Idrees Adhi

**Affiliations:** 1 Chairman Department of Ophthalmology, Dow University of Health Sciences, Karachi, Pakistan; 2 Medical Students, Dow Medical College, DUHS, 109/2, Popular Avenue, Phase 6, D.H.A, Karachi, Pakistan; 3 Associate Professor Microbiology Dow Medical College, Karachi, Pakistan

**Keywords:** Hepatitis B, Hepatitis C, Cataract

## Abstract

**Background:**

To report the findings of preoperative screening regarding prevalence of Hepatitis B and Hepatitis C in patients presenting for cataract surgery.

**Findings:**

A descriptive study was conducted among 377 patients presenting for cataract surgery to Department of Ophthalmology Unit I, CHK from April 2010 to May 2011. Convenience sampling was done to recruit the participants aged 18 years and above. The patients were screened for Hepatitis B and C infections and findings were recorded on a structured compilation sheet.

The total prevalence of both Hepatitis B and Hepatitis C in preoperative Cataract patients was found to be 49 out of 377(12.99%). Overall, 8 out of 377 (2.1%) patients were HBsAg positive and 42 out of 377 (11.1%) were Anti-HCV positive. Only 1 patient was found with a co-infection with both HBsAg and Anti-HCV positive.

**Conclusions:**

High proportions of Hepatitis B and C are reported among preoperative cataract patients of Karachi. Routine serological screening prior to surgery should be made mandatory so that asymptomatic patients would no longer pose a threat to its spread.

## Background

Hepatitis is described as an infection with swelling and inflammation of the liver that if progresses, may lead to cirrhosis or cancer [1]. Sometimes people contract hepatitis with limited or no symptoms but often it leads to jaundice, anorexia (poor appetite) and diarrhea. Hepatitis is caused by a wide variety of causatives like alcohol, poison and autoimmunity but most cases of hepatitis are reported by viruses [1].

Hepatitis B (HBV) and Hepatitis C (HCV) are one of the viral types of Hepatitis that lead to irritation, inflammation and swelling of the liver capable of causing acute and chronic form of hepatitis [2]. Worldwide 2 billion people have been infected with HBV and 350 million (5-15% of the total cases) are carriers of the virus [3]. According to WHO estimates, HCV prevalence is 3% of world population with 170 million cases. Almost 50% of all cases become chronic carriers at risk of liver cirrhosis and liver cancer [4].

HBV can be contracted through the blood, semen, vaginal fluids, and other body fluids of an infected individual having hepatitis B infection [2]. HCV however, can only be contracted through blood to blood contact [5]. The transmission risk of these diseases is more among patients receiving blood transfusions or injection drug users [6].

Unfortunately, once inflicted, these infections show poor response to the available treatment modalities. Therefore precautionary methods are considered the best way to avoid spreading of this disease. Unlike HCV, several vaccines have been developed for HBV that provide long lasting immunity to individuals [7]. It is the most important precautionary measure of HBV as a vaccinated individual may never contract the infection [8]. Both infections, especially the risk of HCV, can be further avoided by use of disposable syringes, screened blood transfusion, avoidance of sexual abuse, antiseptic shaving and use of proper antiseptic measures in hospitals, clinics and operation theaters [6].

Pakistan is also facing a huge burden of these diseases. Although Pakistan is in a moderate Hepatitis B prevalence area, chronic hepatitis B is still a severe problem with a carrier rate of 3-4% [9,10]. Hepatitis B infected blood or fluids is considered the major source of transmission of the disease in Pakistan [11]. On the other hand, Pakistan is considered among the high risk areas of Hepatitis C with about 10 million Pakistani population is infected with Hepatitis C [12] at a prevalence rate of 4-10% [13]. Blood transfusions is still the major cause of HCV transmission in Pakistan; as a survey of blood banks in the large urban centers of the country showed that only about 25% of them tested blood and blood product donations for HCV to keep the cost down [14].

The prevalence of HBsAg and anti-HCV in hospitalized surgical patients is very high. There is lack of routine serological screening prior to surgery which is one of the factors responsible for increased disease transmission. The major risk factors include re-use of contaminated syringes, surgical instruments and improperly screened blood products [6].

Cataracts are changes in clarity of the natural lens inside the eye that gradually degrade visual quality [15].Cataracts develop for a variety of reasons, including long-term exposure to ultraviolet light, exposure to radiation, secondary effects of diseases such as diabetes, and many more. According to a survey conducted in 2007, an estimated 570 000 individuals are bilaterally blind from cataract in Pakistan which is a very significantly high number [16]. There is no non surgical treatment for cataract except some preventive measures. Cataract surgery is the only way of treating this disease. Operation to remove cataracts can be performed at any stage of their development. Nowadays cataract operation can be performed anytime depending upon the visual requirements of the patient [17]. However, because all surgery involves some risk, it is usually worth waiting until there is some change in vision before removing the cataract [17].

The aim of our current study was to estimate the prevalence of Hepatitis B and Hepatitis C among preoperative cataract patients of Karachi, Pakistan. We hypothesized that the prevalence of Hepatitis B and Hepatitis C would be higher than 5% among preoperative cataract patients.

## Methods

The study was approved by Ethical review Committee of Dow University of Health Sciences. All subjects’ information was kept confidential and written informed consent was obtained.

It was a descriptive study based on a survey in Department of Ophthalmology, Unit I Civil Hospital Karachi. All patients admitted for cataract operation from 1st April 2010-1st May 2011 were screened for Hepatitis B and Hepatitis C infections. Out of the intended sample size of 400 screened cataract patients, 377 patients agreed to participate in the study while 23 patients refused consent later on for various reasons like patient denial. Thus the original sample size of 400 patients could not be completed.

Data was collected on the patients’ clinical history and co-morbidities. The findings were recorded in a structured compilation sheet and analyzed through application of statistical tools of analysis. A descriptive statistical analysis was carried out on SPSS 17 software.

All patients of 18 years and above that presented themselves with cataract at the Department of Ophthalmology were included in the study. Patients below 18 years or having any other eye operation besides cataract like glaucoma were excluded from the study.

Rapid chromatography immunoassay for qualitative detection of surface antigen of hepatitis B and antibodies for hepatitis C was the screening technique used in the study. Results that were found positive on screening test were confirmed by ELISA (Enzyme-Linked Immunosorbent Assay) method on (Evolus –4th generation ELISA).

## Findings

A total of 377 out of the intended sample of 400 patients were included in the study. 23 participants refused consent.

Subjects comprised of 200(53.1%) males and 177(46.9%) females with a mean age of 54.16. The total prevalence of both Hepatitis B and Hepatitis C in preoperative Cataract patients was found to be 12.99% (49/377) with Hepatitis C having a higher prevalence of 11.1% (42/377) in comparison to Hepatitis B prevalence of 2.1% (8/377).

14.5% (29/200) of the total male population and 11.3% (20/177) of the total female population were positive for Hepatitis B and Hepatitis C infections.

Male predominance in the total number of positive cases was 59.18% (29/49) in comparison to females with 40.82% (20/49). Only one female patient was found to have co-infection of both Hepatitis B and Hepatitis C.

Individually, Hepatitis B was more common in males 75% (6/8) than females 25% (2/8). Similarly Hepatitis C was also common in males with 54.75% (23/42) in comparison to females 45.25% (19/42) (Table 1).

**Table 1:** Representating data of our study

	**MALE**	**FEMALE**	**TOTAL**
NO. OF SUBJECTS	200	177	377
POSITIVE HEPATITIS B CASES	6	2*	8
POSITIVE HEPATITIS C CASES	23	19*	42
PREVALENCE OF POSITIVE CASES PER POPULATION	14.5%	11.3%	12.99%

*One female had a co-infection of both Hepatitis B and Hepatitis C.

## Discussion

Hepatitis B and Hepatitis C are global diseases that are endemic in many countries. Hepatitis C, in combination with hepatitis B, accounts for 75% of all cases of liver disease around the world [18]. Many Asian countries are highly endemic to both Hepatitis B and Hepatitis C [3,19].

Pakistan, like many developing countries, has a high incidence of Hepatitis B and Hepatitis C of 10% and 4-7% respectively [20]. The prevalence is continuing to rise [21] and in certain parts, especially in the rural areas, the percentage of infected individuals is significantly higher than those reported [22,23].

The total prevalence of Hepatitis B and Hepatitis C in this study was found to be 12.99%. This was higher in comparison to Iftikhar et al found to be 5.75% [24]. However, Khan et al, showed a double prevalence of 24% on eye camps in rural Sindh, rather than hospitalized patients [20]. Ali et al reported HBV prevalence of 3.89% ±1.004% in Pakistani ophthalmic patients [25]. The higher prevalence in eye camps in comparison to hospitalized patients may be due to the fact that the study was conducted in rural areas and had a longer duration of time. Our study comprised of a mixed population of urban population of Karachi, periphery of Karachi and rural areas of Sindh and Baluchistan.

Hepatitis B and Hepatitis C prevalence in preoperative cataract patients was found to be higher in males (59.18%) than females (40.82%). Iftikhar et al also showed that the total prevalence of Hepatitis B and Hepatitis C in males was very high compared to females among preoperative cataract patients of DI Khan [24]. In other different studies done in the country, similar findings and results were seen where males predominated females [26-29]. The reason may be attributed to the fact that males in Pakistan have a higher social mobility and freedom, especially in rural areas as compared to females. Thus they have a greater chance of contracting the infection.

Some studies however contradicted the result, with females having a higher prevalence than males [30,31].

A study conducted in 2010 on different eye camps in Pakistan showed that 108 out of 437 patients were infected with Hepatitis B and Hepatitis C with a higher prevalence of the diseases in females with 60.18% (65/108) than in males with 39.81% (43/108) [20].

The highest number of positive cases of Hepatitis B and Hepatitis C in our study, were found in age group 50-85. As far as cataract patients are concerned, Iftikhar et al also had similar results where both hepatitis B and C were seen to be highly prevalent in the age group between 55-64 years [24].

A study done only on Hepatitis C prevalence in eye camps in Pakistan in 2008, showed that incidence increased as the age increased in study patients. Hepatitis C prevalence was 42.3% in less than 60 years of age to 57.7% in more than 60 years of age [32]. High incidence of Hepatitis C was also seen in a similar study in Japan [33]. Age group 18-30 had the least positive cases comparable to the D.I Khan study where prevalence of HBV and HCV infections was also least in the age group 25-34 years [24].

Age-related cataract is responsible for 48% of world blindness, which represents about 18 million people, according to the World Health Organization (WHO) [33]. Pakistan also has a higher incidence of cataract patients [16]. The higher prevalence of Hepatitis B and Hepatitis C in older age groups can be due to the fact that the incidence of age related cataract is very common. A Japanese study supported this by concluding that Hepatitis C may play a part in older people for developing cataracts [34].

There were however a number of limitations to our study. Hepatitis B and Hepatitis C prevalence in children of Pakistan has seen a major rise in the past few years [35]. However our study was not conducted on participants below 18 years. Because this study was conducted among patients of certain age (and certain age related disease = cataract) the prevalence we found could be over or underestimated. Another limitation of this study is because of the study design other risk factors such as medical care related factors (previous surgeries) drug use, risky sexual behavior, tattooing etc which can influence the presence of hepatitis infection were not obtained. Thus we could only speculate about the age and gender differences in the prevalence. Further studies are needed to address these issues.

With such a higher prevalence of the viral diseases in the population, it is important that patients be regularly screened for Hepatitis B and Hepatitis C. Media should be used as a tool to spread awareness in the public.

## Conclusion

No study is available on hepatitis B and C infection among preoperative cataract patients in Karachi. The aim of the present study was to assess the prevalence of HBV and HCV infection among preoperative cataract patients in Karachi. Routinely screening of preoperative cataract patients are still not carried out. Awareness among doctors to carry out such screening of preoperative patients is necessary for patients’ safety. We hope that this study will prove a stepping stone to implement screening of Hepatitis B and Hepatitis C among preoperative patients.

## Competing interests

The authors declare that they have no competing interests.

## Author’s contributions

SSN: wrote article and decided topic. EUS: wrote article and decided the topic. ANK: Did data collection and wrote paper. STK: Did data collection. FE: Help review manuscript and make changes. IA: Help review manuscript and make changes. All authors read and approved the final manuscript.
